# Cascaded adaptive model predictive and PID control for integrated LFC–AVR enhancement

**DOI:** 10.1038/s41598-026-45726-4

**Published:** 2026-04-18

**Authors:** Mohamed Ayman, Mahmoud A. Attia, Ahmed M. Asim

**Affiliations:** Department of Electrical Power and Machines, Faculty of Engineering, Ain Shams University, Cairo, Egypt

**Keywords:** Load–frequency control, Automatic voltage regulator, Adaptive model predictive control, PID, Energy science and technology, Engineering

## Abstract

Load Frequency Control (LFC) and Automatic Voltage Regulation (AVR) are traditionally designed and tuned separately, which may lead to degraded dynamic performance when power systems experience simultaneous load disturbances and parameter uncertainties. Moreover, fixed-gain controllers often fail to maintain fast and well-damped responses under operating-point variations in both single-area and two-area systems. To address these challenges, this paper proposes a coordinated LFC–AVR control scheme based on a cascaded architecture, in which an adaptive model predictive controller (AMPC) serves as the outer loop, while a PID controller operates in the inner loop to enhance implementability and tracking speed. The AMPC is updated online using recursive least squares (RLS) identification combined with a time-varying Kalman filter (TVKF) to estimate system states and cope with time-varying dynamics. The proposed AMPC–PID strategy is evaluated on single-area and two-area power system models under step load perturbations and significant parameter variations. Its performance is benchmarked against a recent EDO-PID approach, as well as an HS-optimized PID for the AVR loop. Simulation results demonstrate improved damping, reduced under/overshoot, and shorter settling times for both frequency and voltage deviations, confirming the robustness and effectiveness of the coordinated AMPC–PID control strategy under different operating conditions.

## Introduction

The rapid expansion of interconnected power systems has intensified the need to maintain stable and reliable operation. Among the most critical operational requirements are frequency and voltage regulation, as they reflect the balance of active and reactive power in the system. Load Frequency Control (LFC) aims to suppress frequency deviations and maintain scheduled tie-line power exchanges, whereas the Automatic Voltage Regulator (AVR) preserves acceptable voltage levels through excitation control. Importantly, these dynamics are inherently coupled through the turbine–generator–exciter structure, meaning that disturbances affecting active power and rotor speed can influence voltage behavior, while excitation changes can also impact electromechanical dynamics^1–4^.

Despite this inherent coupling, conventional control designs often treat LFC and AVR as independent subsystems. This separation can lead to unsatisfactory transient performance, including larger under/overshoots and longer settling times, particularly under large load disturbances and parameter uncertainties^3–5^. Consequently, coordinated LFC–AVR control strategies have become increasingly important for ensuring well-damped and robust dynamic responses in modern interconnected power systems^5^.

From a practical implementation perspective, Proportional–Integral–Derivative (PID) controllers remain dominant in LFC and AVR applications due to their simplicity, reliability, and ease of deployment. However, fixed-gain PID controllers exhibit limited robustness under operating-point variations, nonlinearities, and parameter uncertainties. To address these limitations, numerous metaheuristic optimization techniques, such as Genetic Algorithm (GA), Particle Swarm Optimization (PSO), Differential Evolution (DE), and Harmony Search (HS), have been widely employed to optimally tune PID gains for improved transient and steady-state performance^6–9^. Nevertheless, these tuning approaches are typically performed offline, which limits the controller’s ability to adapt to real-time changes in system dynamics.

To overcome the limitations of fixed and offline-tuned controllers, Model Predictive Control (MPC) has emerged as a promising advanced control technique. MPC can explicitly handle multivariable interactions, system constraints, and predictive optimization within a unified framework^10^. By utilizing future predictions of system behavior, MPC often achieves superior dynamic performance compared to classical control methods. Furthermore, Adaptive Model Predictive Control (AMPC) enhances these capabilities by updating the prediction model online, thereby improving robustness against uncertainties, load variations, and modeling inaccuracies^11^.

Despite these advantages, most existing MPC and AMPC applications in power systems have focused primarily on frequency regulation, with relatively limited attention given to fully coordinated LFC–AVR control. Moreover, the direct implementation of MPC-based controllers can be constrained by computational complexity and practical implementation considerations. To address these challenges while maintaining high performance, cascaded control structures have been proposed as an effective solution. In such configurations, an advanced predictive controller operates in the outer loop to provide optimal reference signals, while a conventional PID controller in the inner loop ensures fast response and practical implementability^12^.

Recent studies have also explored predictive and cascaded control strategies for renewable-integrated and multi-source power systems. For instance, Kumar et al. developed a Harris Hawks Optimization (HHO)–based MPC for stochastic wind-integrated multi-area power systems to achieve coordinated voltage and frequency regulation under uncertainties. Similarly, Kumar et al. proposed a predictive optimal control strategy to enhance coordinated regulation in renewable-rich interconnected systems. In addition, Ahmad et al. introduced an enhanced frequency–voltage control scheme using capacitive energy storage (CES) combined with an SSA-optimized cascade TID–FOPTID controller, demonstrating the effectiveness of cascade architectures and energy storage in improving disturbance rejection. Although these approaches provide improved dynamic performance, they generally rely on offline-tuned parameters and do not consider an adaptive MPC–PID cascaded framework with online model and state updating for coordinated LFC–AVR enhancement in both single-area and two-area benchmark systems^22–24^.

Motivated by these limitations, this paper proposes a coordinated LFC–AVR control scheme based on a cascaded Adaptive Model Predictive Control (AMPC) and PID architecture. In this structure, the AMPC outer loop is updated online to accommodate time-varying system dynamics, while the inner PID loop ensures fast tracking performance and practical implement ability. The proposed approach is evaluated on both single-area and two-area power system models and benchmarked against optimized PID-based methods. Simulation results demonstrate significant improvements in damping characteristics, transient response, and robustness underload disturbances and parameter variations.

### Contribution of this paper

The main contributions of this work are summarized as follows:

- Development of an integrated LFC–AVR control framework that explicitly accounts for frequency–voltage coupling.
- Design of a cascaded AMPC–PID controller that combines adaptive predictive optimization with fast inner-loop regulation to improve dynamic performance and robustness.
- Comprehensive comparative evaluation against recent optimized PID-based approaches to demonstrate the effectiveness of the proposed method.
- Validation of the proposed control strategy on both single-area and multi-area power system models under various load disturbances and significant parameter uncertainties.

The remaining parts of the paper is organized as follows: Section 2 presents the proposed controller, Section 3 describes the system models, Section 4 discusses simulation results, and Section 5 concludes the paper.

## Proposed adaptive model predictive controller (AMPC)

### Overview and motivation

In many practical applications, system parameters vary over time due to nonlinearities, load changes, or environmental conditions. A conventional Model Predictive Controller (MPC) assumes a fixed linear model, which can lead to poor control performance when the real plant deviates from this model. The proposed Adaptive Model Predictive Controller (AMPC) is designed to overcome this limitation by continuously updating the prediction model according to the current operating conditions. This allows the controller to maintain accurate predictions, satisfy system constraints, and achieve robust performance under parameter variations^13,14^.

### Time-varying state-space model

The AMPC framework uses a discrete-time, linear time-varying (LTV) state-space representation of the plant as follows in Equations (1) and (2)^15^:

1 $$\mathrm{x}\left(\mathrm{k}+1\right)={\mathrm{A}}_{\mathrm{k}}\mathrm{x}\left(\mathrm{k}\right)+{\mathrm{B}}_{\mathrm{k}}\mathrm{u}\left(\mathrm{k}\right)+{\mathrm{E}}_{\mathrm{k}}\mathrm{d}\left(\mathrm{k}\right)$$

2 $$\mathrm{y}(\mathrm{k})={\mathrm{C}}_{\mathrm{k}}\mathrm{x}(\mathrm{k})$$

where

- $x(k)$ is the state vector,
- $u(k)$ is the control input,
- $d(k)$ represents measurable or unmeasurable disturbances,
- $y(k)$ is the plant output
- The matrices ${A}_{k},{B}_{k},{C}_{k},{E}_{k}$ are updated at each sampling instant to reflect the current dynamics of the plant.

The model parameters are identified online using recursive estimation techniques (e.g., Recursive Least Squares or Extended Kalman Filter) to capture time-varying system behavior. Only the latest model $\left({A}_{k},{B}_{k},{C}_{k}\right)$ is used during the current prediction horizon, following the receding-horizon principle of MPC^16^.

### Online RLS-based system identification

To enable continuous adaptation of the predictive model, the plant parameters are identified online using the Recursive Least Squares (RLS) algorithm. The RLS estimator updates a parameter vector $\theta (k)$ that represents the dominant coefficients of the governor–turbine–generator subsystem. The algorithm follows the standard recursive update equations (3),(4),(5) and (6)^15,16^:

3 $$\mathrm{e}(\mathrm{k})=\mathrm{y}(\mathrm{k})-{\rm{\varphi }}^{\mathrm{T}}(\mathrm{k})\uptheta (\mathrm{k}-1)$$

4 $$\mathrm{K}(\mathrm{k})=\frac{\mathrm{P}\left(\mathrm{k}-1\right)\rm{\varphi }\left(\mathrm{k}\right)}{\uplambda +{\rm{\varphi }}^{\mathrm{T}} \left(\mathrm{k}\right)\mathrm{P}\left(\mathrm{k}-1\right)\rm{\varphi }\left(\mathrm{k}\right)}$$

5 $$\uptheta (\mathrm{k})=\uptheta (\mathrm{k}-1)+\mathrm{K}(\mathrm{k})\mathrm{e}(\mathrm{k})$$

6 $$\mathrm{P}(\mathrm{k})=\frac{1}{\uplambda }[\mathrm{P}(\mathrm{k}-1)-\mathrm{K}\left(\mathrm{k}\right){\rm{\varphi }}^{\mathrm{T}}(\mathrm{k})\mathrm{P}(\mathrm{k}-1)]$$

The forgetting factor $\lambda$ typically ($0.98\le \lambda \le 1$) determines how fast the estimator tracks time-varying parameters. The identified parameter vector is mapped to the time-varying state-space matrices ($A(k),B(k),C(k)$), which are updated at each sampling instant to provide the AMPC with an accurate prediction model^16^. Parameter bounding and covariance resetting are applied to enhance robustness under noise and sudden variations^16^.

### Nominal deviation form

For constraint handling, the plant model is written in deviation form with respect to a nominal operating point $({x}_{0},{u}_{0},{y}_{0})$ as in equations (7) and (8):

7 $$\rm{\Delta x}(\mathrm{k}+1)={\mathrm{A}}_{\mathrm{k}}\rm{\Delta x}(\mathrm{k})+{\mathrm{B}}_{\mathrm{k}}\rm{\Delta u}(\mathrm{k})$$

8 $$\rm{\Delta y}(\mathrm{k})={\mathrm{C}}_{\mathrm{k}}\rm{\Delta x}(\mathrm{k})$$

Where $$\rm{\Delta x}\left(\mathrm{k}\right)=\mathrm{x}\left(\mathrm{k}\right)-{\mathrm{x}}_{0}, \Delta u\left(k\right)=u\left(k\right)-{u}_{0},$$ and $$\Delta y(k)=y(k)-{y}_{0}.$$ 

This formulation simplifies the control optimization problem and ensures that the controller reacts only to deviations from nominal conditions^15^.

### Receding-horizon optimization

At each sampling instant k, the AMPC computes a control sequence that minimizes a finite-horizon cost function as in equation (9):

9 $$\mathrm{J}={\sum }_{\mathrm{i}=1}^{\mathrm{Np}}\parallel \mathrm{y}\left(\mathrm{k}+\mathrm{i}\right)-\mathrm{r}\left(\mathrm{k}+\mathrm{i}\right){\parallel }_{\mathrm{Q}}^{2}+{\sum }_{\mathrm{i}=0}^{\mathrm{Nc}-1}\parallel \rm{\Delta u}\left(\mathrm{k}+\mathrm{i}\right){\parallel }_{\mathrm{R}}^{2}$$

where ${N}_{p}$ is the prediction horizon, ${N}_{c}$ is the control horizon, $Q$ and $R$ are weighting matrices, and $r(k)$ is the reference signal.

The optimization is subject to the following constraints such as:

$${\mathrm{u}}_{min} \le \mathrm{u}\left(\mathrm{k}+\mathrm{i}\right)\le {\mathrm{u}}_{max} ,\rm {\mathrm{u}}_{min}\le \rm{\Delta u}\left(\mathrm{k}+\mathrm{i}\right)\le\Delta {\mathrm{u}}_{max} , {\mathrm{y}}_{min}\le \mathrm{y}(\mathrm{k}+\mathrm{i})\le {\mathrm{y}}_{max}$$

Only the first control move $u(k)$ is applied, while the optimization problem is solved again at the next time step using the updated plant model (receding-horizon principle)^13,17^.

### State estimation (Time-varying Kalman filter)

Since all system states are not directly measurable, the AMPC uses a linear time-varying Kalman filter (LTVKF) to estimate them. At each time step k, the filter updates the state estimate $x(k)$ and the covariance matrix $P(k)$ according to the current plant model^14^.

The key recursive equations are:

1. Prediction step as in equation (10):
  10 $${\mathrm{P}}_{\mathrm{k}|\mathrm{k}-1}={\mathrm{A}}_{\mathrm{k}}{\mathrm{P}}_{\mathrm{k}-1|\mathrm{k}-1}{\mathrm{A}}_{\mathrm{k}}^{\mathrm{T}}+\mathrm{Q}$$
2. Kalman gains as in equations (11) and (12):
  11 $${\mathrm{L}}_{\mathrm{k}}= \left({\mathrm{A}}_{\mathrm{k}}{\mathrm{P}}_{\mathrm{k}|\mathrm{k}-1} {\mathrm{C}}_{\mathrm{k}}^{\mathrm{T}}+\text{ N}\right){\left({\mathrm{C}}_{\mathrm{k}}{\mathrm{P}}_{\mathrm{k}|\mathrm{k}-1} {\mathrm{C}}_{\mathrm{k}}^{\mathrm{T}}+\text{ R}\right)}^{-1}$$
  12 $${\mathrm{M}}_{\mathrm{k}} = {\mathrm{P}}_{\mathrm{k}|\mathrm{k}-1} {\mathrm{C}}_{\mathrm{k}}^{\mathrm{T}} {\left({\mathrm{C}}_{\mathrm{k}} {\mathrm{P}}_{\mathrm{k}|\mathrm{k}-1} {\mathrm{C}}_{\mathrm{k}}^{\mathrm{T}} +\text{ R}\right)}^{-1}$$
3. Covariance update as in equation (13):
  13 $${\mathrm{P}}_{\mathrm{k}+1|\mathrm{k}} = {\mathrm{A}}_{\mathrm{k}} {\mathrm{P}}_{\mathrm{k}|\mathrm{k}-1} {\mathrm{A}}_{\mathrm{k}}^{\mathrm{T}} - {\left({\mathrm{A}}_{\mathrm{k}} {\mathrm{P}}_{\mathrm{k}|\mathrm{k}-1} {\mathrm{C}}_{\mathrm{k}}^{\mathrm{T}} +\text{ N}\right)\mathrm{L}}_{\mathrm{k}}^{\mathrm{T}} +\text{ Q}$$

Here, $Q,R$ and $N$ are covariance matrices representing process noise, measurement noise, and cross-correlation, respectively. When the model remains constant, the LTVKF converges with the traditional steady-state Kalman filter used in standard MPC^14,16^.

### AMPC strategy

The AMPC maintains recursive feasibility and closed-loop stability by updating model parameters gradually and ensuring constraint satisfaction at all times.

Robustness is achieved by:

- Applying tightened constraint sets around the nominal trajectory^15^.
- Monitoring model parameter changes and trigger conservative fallback control if large deviations occur^16^.
- Including terminal costs and stability constraints in the optimization problem^17^.

This ensures stable operation even under significant system variations and modeling uncertainty.

### Implementation steps of the adaptive control strategy

The complete implementation of the proposed AMPC strategy follows the steps below:

#### Initialization

The initial state-space model $({A}_{0}, {B}_{0}, {C}_{0})$, RLS parameters ${\theta }_{0}$, forgetting factor $\lambda$, and the MPC horizons (${N}_{p}$,${N}_{c}$) are initialized at $k=0$.

#### State measurement

At each sampling instant, the measurable outputs (frequency deviation and tie-line power deviation) are fed back to the controller.

#### Online identification (RLS)

The RLS estimator updates the parameter vector $\theta (k)$ and regenerates the time-varying state-space matrices $(A\left(k\right),B\left(k\right),C\left(k\right))$.

#### State estimation

The updated model is used by the time-varying Kalman filter to obtain the estimated states $x(k)$.

#### MPC optimization

Using the updated model, the AMPC solves the constrained finite-horizon optimization problem and computes the optimal control sequence.

#### Control application

Only the first element of the optimal sequence is applied to the plant (“receding horizon”), ensuring closed-loop operation.

#### Model update

Steps 2–6 repeat at every sampling instant, allowing the controller to adapt to parameter variations, disturbances, and nonlinear operating conditions.

This implementation ensures real-time adaptability and explains how the proposed controller differs fundamentally from conventional MPC and fixed-gain PI/PID strategies^15,16^.

### AMPC tuning procedure

In the proposed cascaded control structure, the Adaptive Model Predictive Controller (AMPC) is employed as an outer-loop controller, while a conventional PID controller is used in the inner loop to ensure fast local regulation. The tuning of the AMPC parameters, namely the prediction horizon ${N}_{p}$, control horizon ${N}_{c}$, and weighting matrices $Q$ and $R$, was carried out following a systematic procedure commonly adopted in adaptive MPC applications^14^. Several candidate parameter sets were evaluated under step and time-varying load disturbances.

The final AMPC parameters were selected based on minimizing the Integral of Time-weighted Absolute Error (ITAE) criterion, while maintaining an appropriate trade-off between transient performance, control effort, and robustness. The cascaded PID controller was tuned separately using a trial-and-error approach, aiming to achieve fast inner-loop dynamics without introducing excessive overshoot or interaction with the outer-loop AMPC. This tuning strategy ensures that the PID controller effectively tracks the reference signal generated by the AMPC, while preserving the overall stability of the cascaded structure.

The separation between the adaptive predictive outer loop and the conventional inner-loop PID allows the proposed controller to combine the adaptability and predictive capabilities of AMPC with the simplicity and fast response characteristics of PID control, resulting in improved overall system performance.

### Cascaded AMPC and PID integration with combined LFC-AVR

The adaptive model predictive controller (AMPC) is implemented and integrated into the combined LFC–AVR system using MATLAB/Simulink within a closed-loop control configuration. In the proposed cascaded architecture, the AMPC operates in the outer loop, where it generates the reference signal for the inner-loop PID controller based on the measured system outputs and desired operating points. The controller continuously receives the measured plant output ($mo$), and reference signal ($ref$), representing the actual system response and the target operating conditions, respectively.

An external function block, labeled “Update Plant Model”, is employed to provide the AMPC with time-varying state-space matrices ${A}_{k}$, ${B}_{k}$, ${C}_{k}$, and ${D}_{k}$, which describe the current linearized dynamics of the power system. This adaptive mechanism enables the AMPC to update its internal prediction model at each control interval, allowing it to accurately capture changes in system operating conditions, nonlinear behavior, and parameter variations.

Using the updated prediction model, the AMPC computes the optimal control signal by minimizing the predefined cost function while satisfying the control objectives. The resulting control action is then passed to the inner-loop PID controller, which ensures fast and smooth regulation of the plant. The PID output is subsequently applied to the power system model, forming a cascaded feedback loop that effectively compensates for load disturbances and dynamic uncertainties.

The disturbance inputs and frequency deviation outputs incorporated in the Simulink model allow continuous monitoring of system performance and facilitate real-time adaptive correction. This cascaded AMPC–PID configuration ensures stable operation and high-quality transient performance for the combined LFC–AVR system. Compared to standalone MPC or fixed-parameter PID controllers, the proposed adaptive cascaded structure offers enhanced robustness, improved tracking accuracy, and superior disturbance rejection capabilities.

## Model under study

The dynamic interaction between the Load Frequency Control (LFC) and Automatic Voltage Regulator (AVR) loops is examined through an integrated Simulink-based model to achieve more accurate transient and steady-state performance analysis. Unlike simplified approaches that treat both loops independently, the adopted modeling framework explicitly accounts for their mutual influence within the overall power system dynamics. The proposed control strategy is evaluated using a single-area power system model, where both mechanical and electrical subsystems are considered simultaneously^18,19^.

The primary objective of the LFC loop is to suppress transient frequency deviations, eliminate steady-state error, and enhance overall system stability by regulating the real power balance. In contrast, the AVR loop maintains the terminal voltage magnitude of the synchronous generator at a specified reference value by adjusting the excitation voltage. Through reactive power control, the AVR contributes significantly to improving steady-state voltage stability. The combined modeling of LFC and AVR allows the investigation of coupling effects between frequency and voltage regulation under dynamic operating conditions. The detailed transfer functions and parameter settings of the LFC and AVR subsystems are summarized in equations (14), (15), (16), (17), (18), (19), (20), and (21)^18,19^.

In many studies, the Load Frequency Control (LFC) and Automatic Voltage Regulator (AVR) loops are assumed to be independent. However, in practical power systems, these loops exhibit noticeable interactions during dynamic conditions. The AVR loop, due to its fast response, regulates the generator excitation and alters the internal electromotive force (EMF), which directly affects the real power output and, consequently, the frequency dynamics governed by the LFC loop.

14 $$\text{Amplifier model }=\frac{{K}_{A}}{ 1+{\tau }_{AS}}$$

15 $$\text{Exciter model }=\frac{{K}_{E}}{ 1+{\tau }_{ES}}$$

16 $$\text{Generator model }=\frac{{K}_{G}}{ 1+{\tau }_{GS}}$$

17 $$\text{Sensor model }=\frac{{K}_{R}}{ 1+{\tau }_{RS}}$$

18 $$\text{Governor model }=\frac{{K}_{g}}{ 1+{\tau }_{gS}}$$

19 $$\text{Turbine model }=\frac{{K}_{T}}{ 1+{\tau }_{TS}}$$

20 $$\mathrm{Inertia}/\text{Load model }=\frac{{K}_{l}}{ 1+{\tau }_{lS}}$$

21 $$\text{Governor frequency bias }=\text{ B}.\text{ D}$$

22 $$\Delta \text{Pe }=\text{ Ps }\Delta\updelta +\text{ K}2\text{ E}^\prime$$

The incremental electrical power deviation resulting from small variations in rotor angle and excitation can be expressed as equation (22):

where ${P}_{s}$ is the synchronizing power coefficient, $\Delta \delta$ denotes the rotor angle deviation, and ${K}_{2}$ represents the sensitivity of electrical power to changes in stator EMF $E^{\prime}$.

Similarly, the terminal voltage deviation is given by equation (23):

23 $$\Delta \text{Vt }=\text{ K}5 \Delta\updelta +\text{ K}6\text{ E}^\prime$$

where ${K}_{5}$ and ${K}_{6}$ describe the effects of rotor angle and stator EMF variations on terminal voltage, respectively.

The internal stator EMF dynamics are modeled as equation (24):

24 $$\mathrm{E}^\prime= \frac{KG}{ 1+\tau G} (\text{Vf }-\text{ K}4\,\Delta\,\updelta )$$

This representation captures the coupling between excitation control and rotor angle variations, enabling accurate modeling of the LFC–AVR interaction.

### Single area power system

**Fig. 1:**
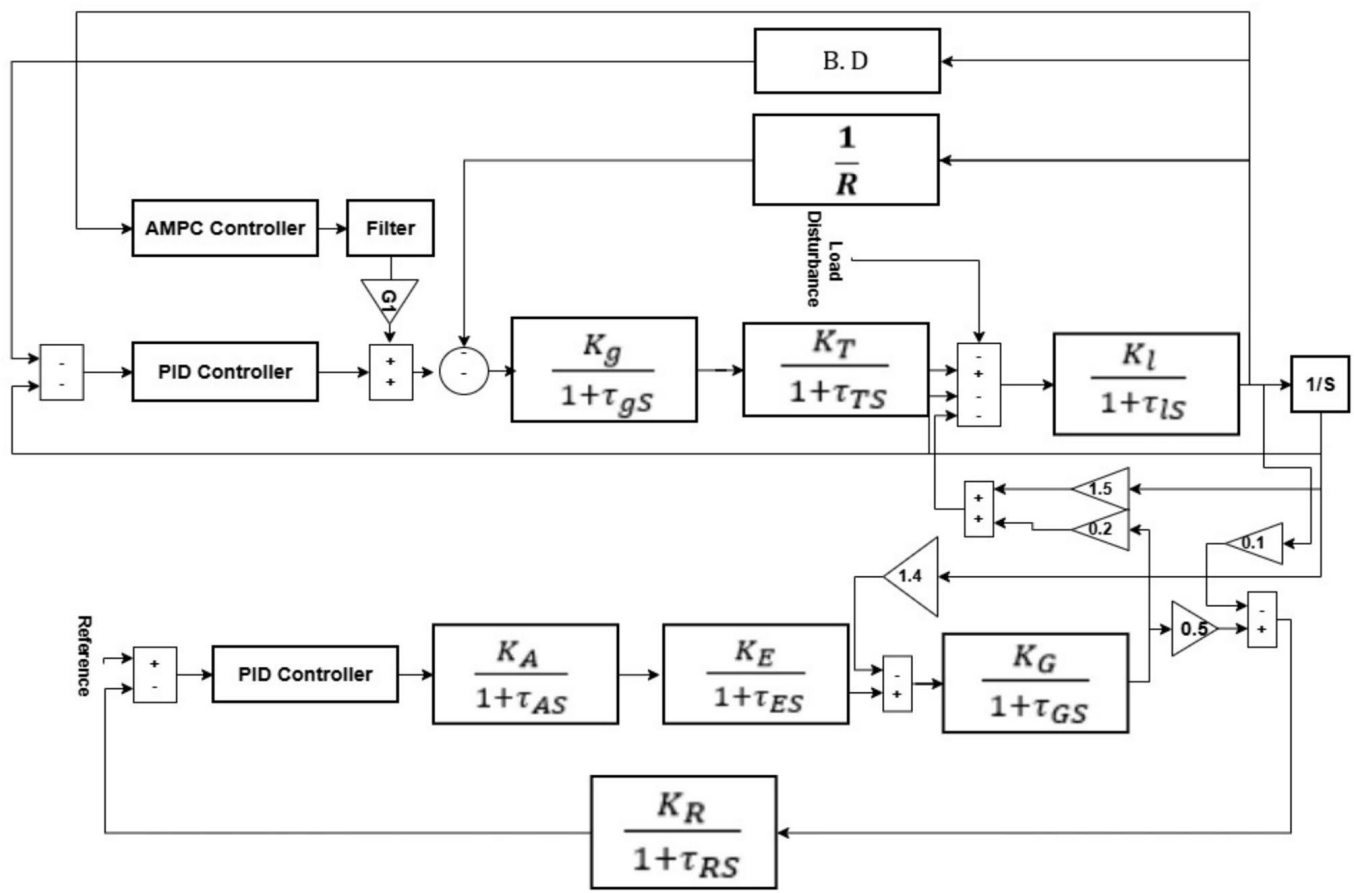
Model of LFC with AVR system with AMPC cascaded with PID controller.

The implementation of the combined LFC and AVR system for a single area model is shown in Fig. 1, which uses an Adaptive model predictive controller (AMPC) cascaded with PID controller to improve the system’s dynamic performance^20^.

### Double area power system

**Fig. 2:**
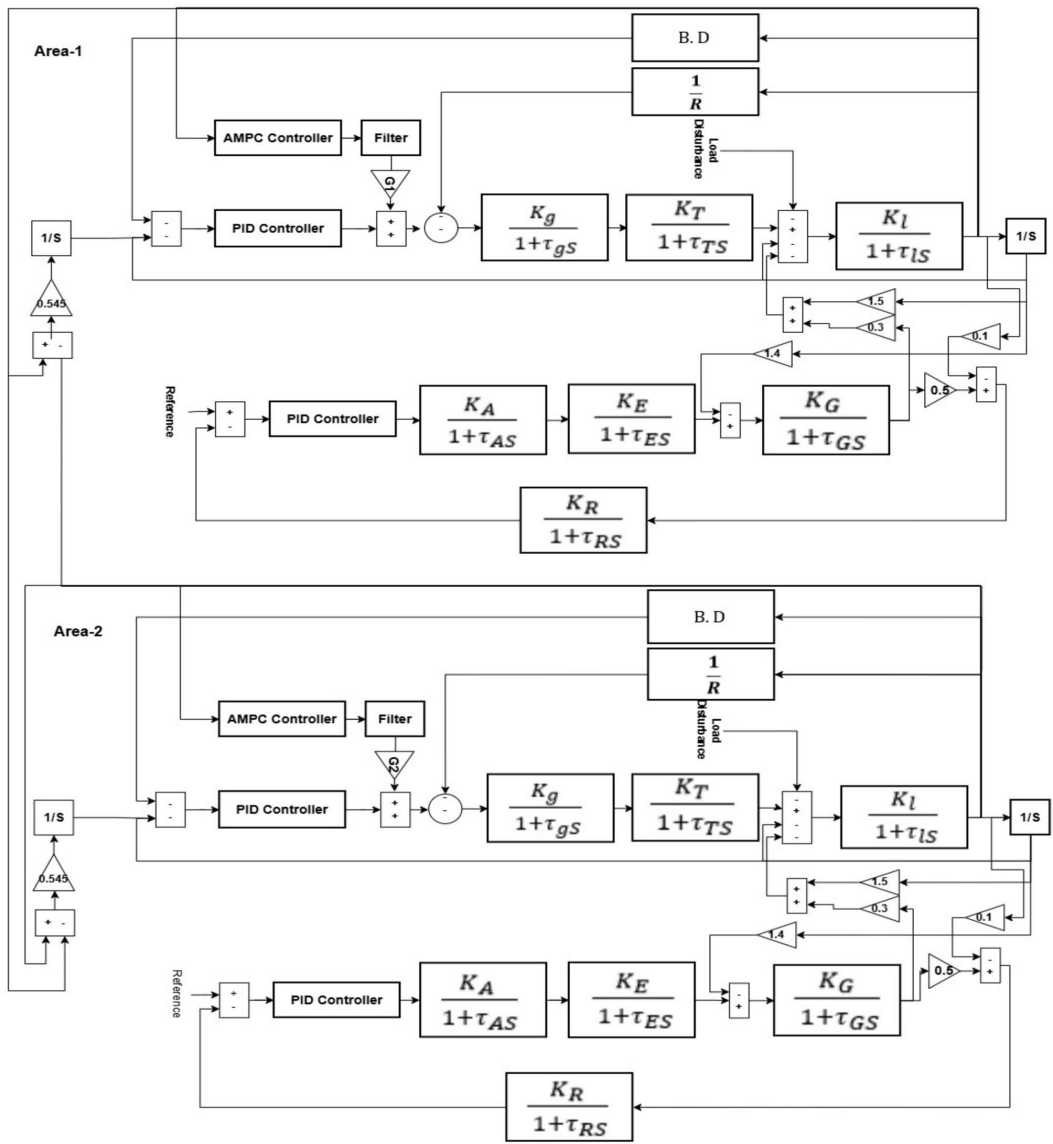
Two-area with combined LFC−AVR.

The two-area interconnected power system model controlled by an adaptive model predictive controller (AMPC) cascaded with PID considered in this study is illustrated on Fig. 2^21^.

## Simulation and results

In this paper, the proposed adaptive model predictive controller (AMPC) cascaded with PID controller are subjected to test their effectiveness in a single area power system and double area power system against different controllers.

### Single area power system

Table 1A illustrates the single area system parameters. Table 1B illustrates the Best-Fit PID Controller Gains for PID controller cascaded with AMPC all gains optimized by trial and error, and according to AVR PID controller gains optimized by Harmony search based on ±10% EDO optimization^21^.

**Table 1:** A: Single-area system parameters^20^. B: Single-area system best-fit controller gains^20^.

Parameter/Gains	Single area parameters
${T}_{g}$	0.2 s
${K}_{g}$	1
${T}_{t}$	0.5 s
${K}_{t}$	1
$R$	0.05
$B$	26
$D$	0.8
$M$	10 s
${K}_{a}$	10
${T}_{a}$	0.1 s
${K}_{e}$	1
${T}_{e}$	0.4 s
${K}_{g}$	0.8
${T}_{g}$	1.4 s
${K}_{s}$	1
${T}_{s}$	0.05s

Parameter/Gains	PIDs gains
${K}_{p1}$	5.7
${K}_{i1}$	4.4
${K}_{d1}$	3.3
${K}_{p2}$	2.7642
${K}_{i2}$	0.5406
${K}_{d2}$	0.7583

#### Results of case study 1

The LFC system is tested using two controllers: The optimized PID controller by exponential distribution optimization using two types of objective functions, ITSE and ITAE^20^ and the proposed Adaptive Model Predictive Controller (AMPC) cascaded with PID controller, The AVR system is tested using two controllers: The optimized PID controller by exponential distribution optimization using two types of objective functions, ITSE and ITAE^20^ and the proposed PID Controller optimized by Harmony Search (HS) optimization based on ±10% from EDO-PID controller gains^20^. A step-load disturbance of 0.2 p.u is applied to evaluate their performance. The PID controller gains, compared with those reported in^20^, show a high degree of consistency, confirming the robustness and accuracy of the simulation, as summarized in Table 1B, which presents the best-fit gains for Case Study 1.

**Fig. 3:**
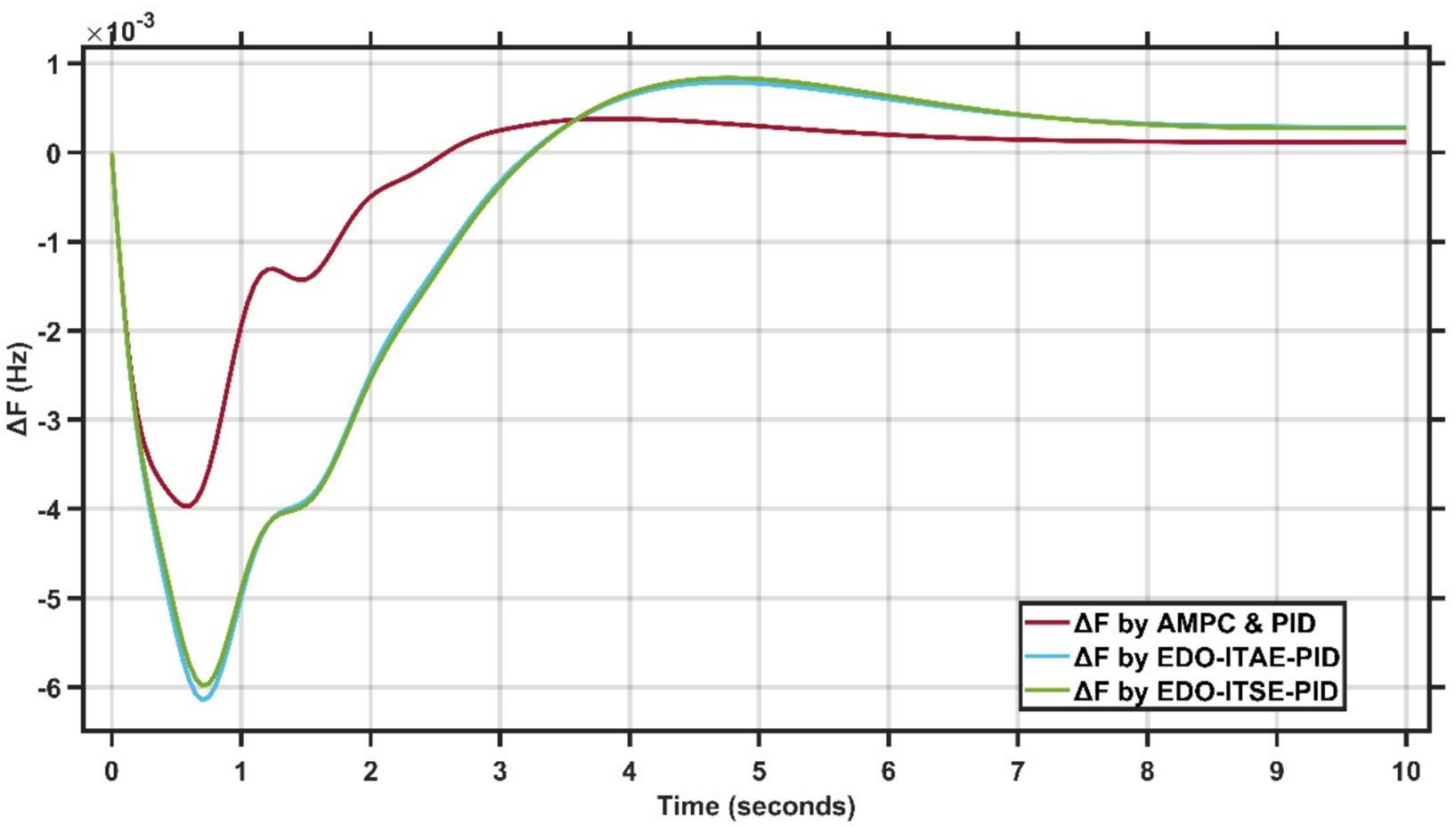
Change in frequency between AMPC cascaded with PID and EDO-PID controller for case study 1.

The transient response specifications of the LFC system for case study #1 are summarized in Table 2. Fig. 3 presents the frequency deviation response of a single-area power system under a step load disturbance.

**Table 2:** Transient response specifications of case study 1.

Variables	AMPC cascaded with PID	EDO-PID controller by ITSE	EDO-PID controller by ITAE
Overshoot	0.3 × 10⁻^3^	0.8 × 10⁻^3^	0.7 × 10⁻^3^
Undershoot	−4 × 10⁻^3^	–6 × 10⁻^3^	–6.2 × 10⁻^3^
Settling time	≈ 4 s	6 s	7 s

From Fig. 3, it is clear that the proposed AMPC with PID controller achieves a maximum undershoot of approximately $-4.0\times {10}^{-3}$ Hz, compared to nearly $-6.0 \times {10}^{-3}$ Hz for both EDO-ITSE-PID and EDO-ITAE-PID, corresponding to an undershoot reduction of about 33%. The positive overshoot is limited to approximately $0.35\times {10}^{-3}$ Hz for the proposed controller, whereas higher overshoots of about $0.8\times {10}^{-3}$ Hz and $0.9\times {10}^{-3}$ Hz are observed for the EDO-based controllers. Moreover, the settling time is reduced to approximately 4 s with the proposed controller, compared to around 6 s and 7 s for the EDO-ITSE-PID and EDO-ITAE-PID controllers, respectively, confirming the superior damping and faster dynamic performance of the proposed AMPC with PID controller.

**Fig. 4:**
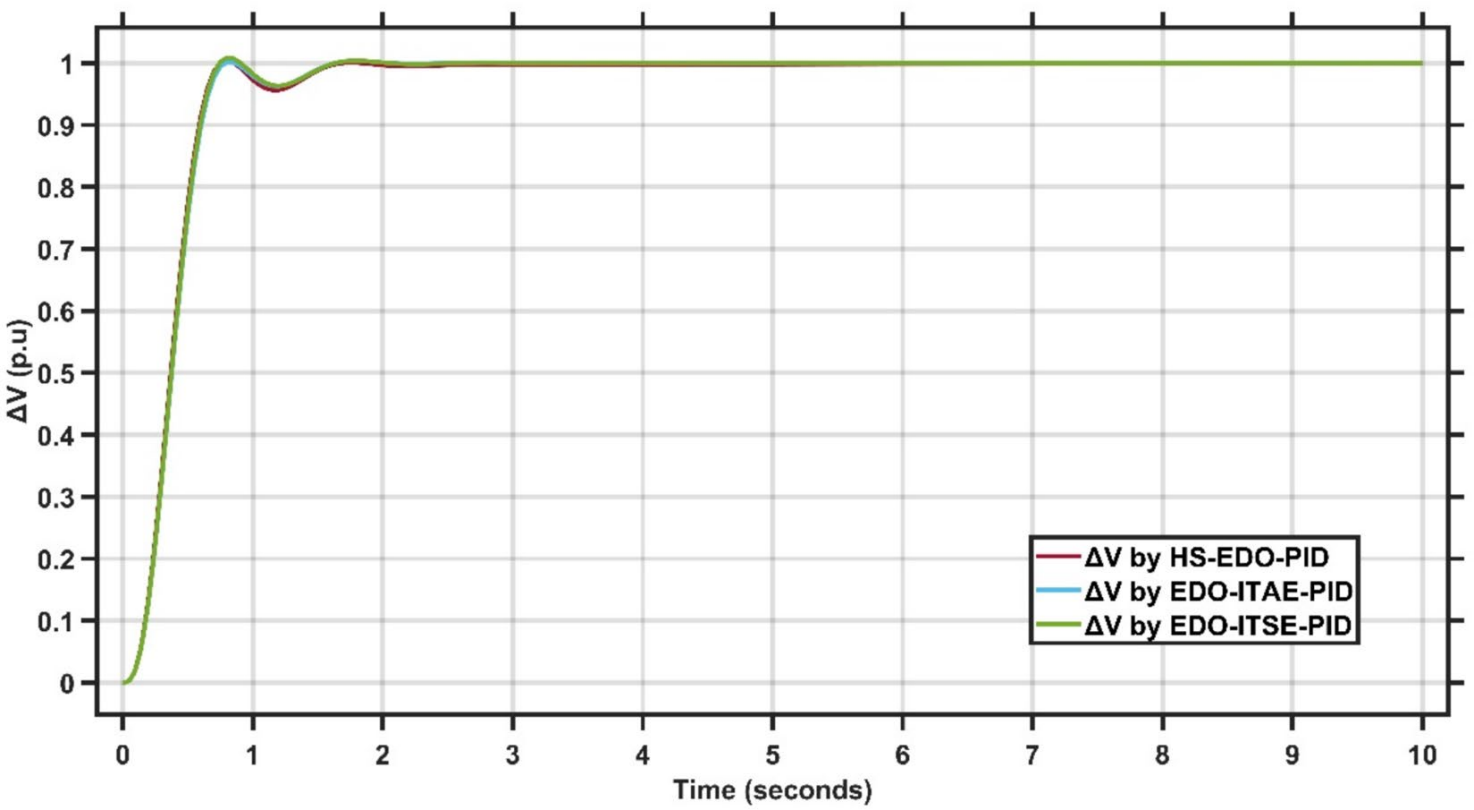
AVR response with HS-EDO-PID and EDO-PID for case study 1.

Figure 4 illustrates the terminal voltage deviation response of the AVR system under a step reference change for different controllers.

From Fig. 4, it is clear that the proposed HS-EDO-ITSE-PID controller achieves a fast voltage rise with a peak overshoot of approximately 1–1.5%, compared to about 2% for the EDO-ITSE-PID and nearly 2.5% for the EDO-ITAE-PID controllers. Moreover, the proposed controller settles to the nominal voltage value in approximately 1.5 s, while the EDO-ITSE-PID and EDO-ITAE-PID controllers require around 2 s and 2.5 s, respectively. The reduced overshoot and faster settling time confirm the enhanced damping and improved transient performance of the HS-EDO-ITSE-PID controller in AVR applications.

#### Robustness analysis

This section evaluates the robustness and reliability of the proposed control schemes applied to the combined LFC–AVR single-area power system. Two complementary robustness tests are conducted to assess controller performance under load disturbances and parameter uncertainties. For the LFC loop, the proposed cascaded AMPC–PID controller is compared with EDO-PID controller, while for the AVR loop, the proposed HS-EDO-PID controller is against the EDO-PID controller.

In the first test, load disturbances are introduced in the form of step changes in load demand with magnitudes of 0.2, 0.3, and 0.4 p.u. These disturbances are applied to evaluate the dynamic response of the combined LFC–AVR system under varying operating conditions. The frequency response obtained using the proposed controllers are compared with its response under different conditions.

**Fig. 5:**
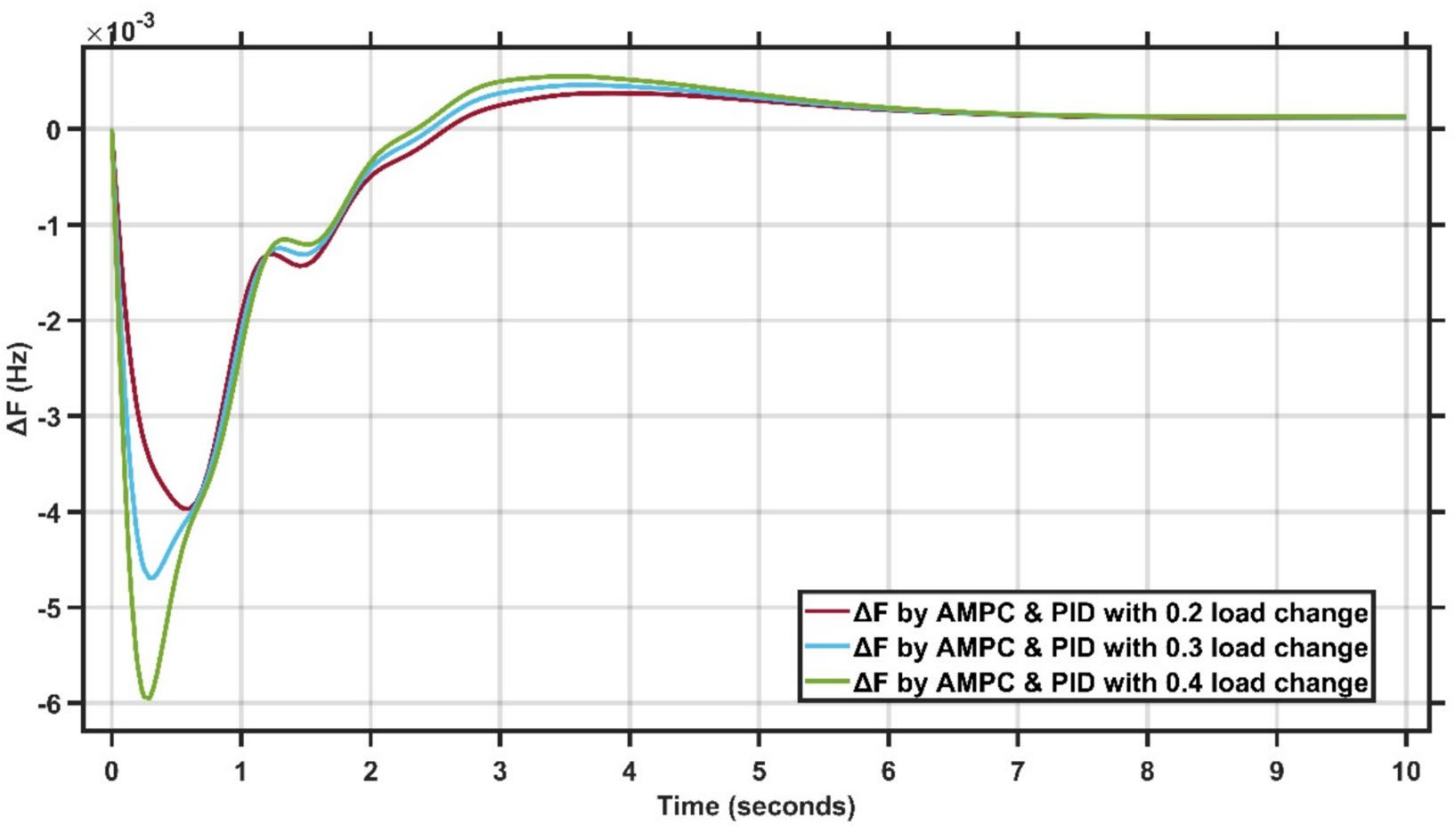
LFC response with Cascaded AMPC and PID under different load change conditions.

Figure 5 presents the frequency deviation response of a single-area power system under different load change conditions.

From Fig. 5, it is clear that the robustness of the proposed AMPC with PID controller under different load disturbances of 0.2, 0.3, and 0.4 p.u. The maximum frequency undershoot increases proportionally with the load change, reaching approximately $-4.0\times {10}^{-3}$ Hz for a 0.2 p.u. disturbance, about $-4.8\times {10}^{-3}$ Hz for 0.3 p.u., and nearly $-6.0\times {10}^{-3}$ Hz for 0.4 p.u. load variation. Despite the increased disturbance magnitude, the system maintains a limited positive overshoot of approximately $0.4-0.6\times {10}^{-3}$ Hz and converges to steady state within about 4–5 s for all cases. These results confirm that the proposed controller preserves stable operation and acceptable dynamic performance under wide load variations, demonstrating strong robust characteristics.

**Fig. 6:**
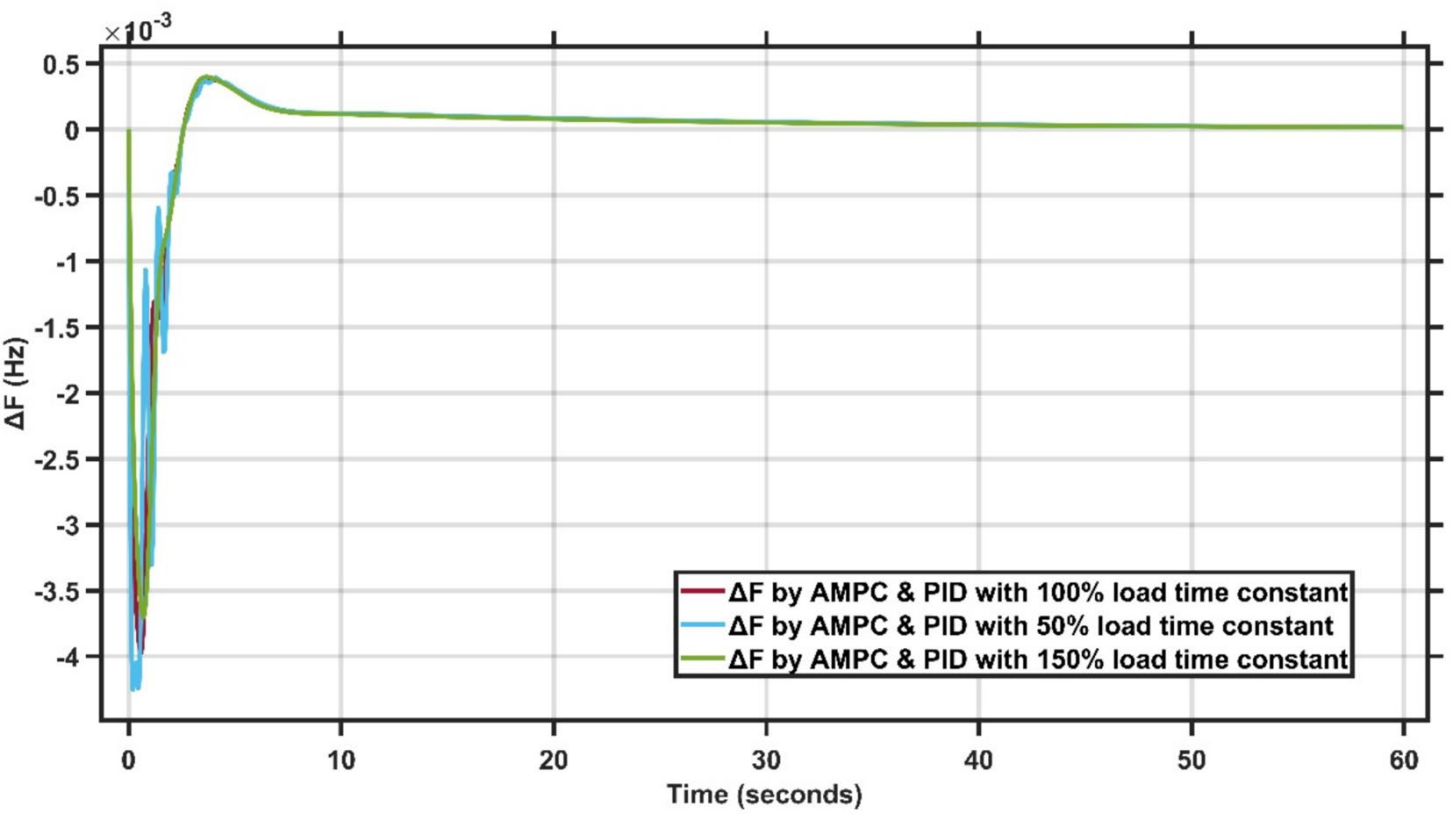
LFC response with cascaded AMPC and PID with variation ±50% time constant.

Figure 6 presents the frequency deviation response of a single-area power system with variation ±50% time constant.

**Fig. 7:**
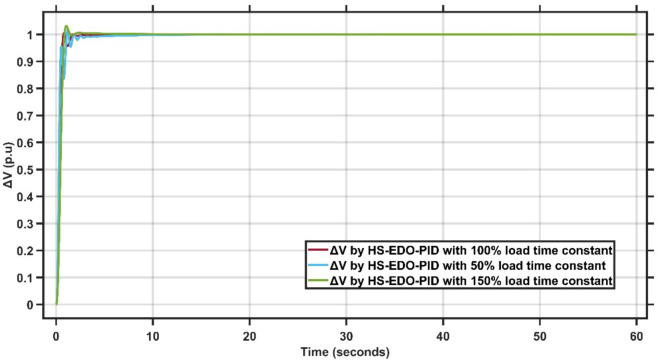
AVR response with HS-EDO-PID with variation ±50% time constant.

From Fig. 6, it is clear that the sensitivity of the proposed AMPC with PID controller to variations in the load time constant at 50%, 100%, and 150% of its nominal value. The maximum frequency undershoot remains bounded, reaching approximately $-4.2\times {10}^{-3}$ Hz for the 50% case, about $-3.8\times {10}^{-3}$ Hz for the nominal 100% condition, and nearly $-3.6\times {10}^{-3}$ Hz for the 150% load time constant. A small positive overshoot of approximately $0.4-0.5\times {10}^{-3}$ Hz is observed in all cases, while the system converges smoothly to steady state within about 8–10 s despite the transient oscillations at early instants. These results confirm that the proposed controller maintains stable and consistent frequency regulation performance under significant variations in load dynamics. Figure 7 illustrates the terminal voltage deviation response of the AVR system with variation ±50% time constant.

From Fig. 7, it is clear that the sensitivity of the AVR terminal voltage deviation under variations of the load time constant at 50%, 100%, and 150% of its nominal value using the proposed HS-EDO-PID controller. The voltage response exhibits a fast rise with a small overshoot of approximately 1–2% for all cases, while the settling time remains nearly unchanged at about 1–1.5 s. Despite noticeable transient oscillations during the initial instants, the voltage converges smoothly to the nominal value without steady-state error. These results confirm that the proposed controller maintains robust voltage regulation performance and strong insensitivity to load time constant variations in AVR applications.

### Double area power system

Table 3A illustrate the double area system parameters. Table 3B illustrate the Best-Fit PID controller gains for PID controller cascaded with AMPC all gains optimized by trial and error, and for AVR system PID controller gains optimized by Harmony search based on ±10% EDO optimization^21^.

**Table 3:** A: Double-area system parameters^20^. B: Double-area system best-fit controller gains^20^.

Parameter/Gains	Area-1 parameters	Area-2 parameters
${T}_{g}$	0.08 s	0.12 s
${K}_{g}$	1	1
${T}_{t}$	0.3 s	0.15 s
${K}_{t}$	1	1
$R$	2.4	1.2
$B$	1	1
$D$	1	1
$M$	20 s	10 s
${K}_{l}$	120	100
${K}_{a}$	10	10
${T}_{a}$	0.1 s	0.1 s
${K}_{e}$	1	1.5
${T}_{e}$	0.4 s	0.6 s
${K}_{G}$	1	1.5
${T}_{G}$	1s	1.5 s
${K}_{s}$	1	1
${T}_{s}$	0.01 s	0.01 s

Parameter/Gains	Area-1 PIDs gains	Area-2 PIDs gains
${K}_{p1}$	5.5	4.5
${K}_{i1}$	6	5
${K}_{d1}$	2	1.3
${K}_{p2}$	1.382340	2.292709
${K}_{i2}$	0.684990	1.099330
${K}_{d2}$	0.422136	0.694060

#### Results of case study 1

The LFC system is tested using two controllers: The optimized PID controller by exponential distribution optimization.[20]and the proposed Adaptive Model Predictive Controller (AMPC) cascaded with PID controller, The AVR system is tested using two controllers: The optimized PID controller by exponential distribution optimization^20^ and the proposed PID Controller optimized by Harmony Search (HS) optimization based on ±10% from EDO-PID controller gains^20^. A step-load disturbance of 0.02 p.u is applied to evaluate their performance. The PID controller gains, compared with those reported in^20^, show a high degree of consistency, confirming the robustness and accuracy of the simulation, as summarized in Table 3B, which presents the best-fit gains for Case Study 1.

**Fig. 8:**
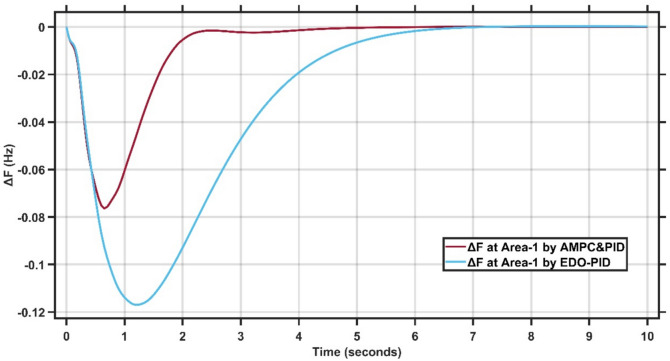
Change in frequency between AMPC cascaded with PID and EDO-PID controller at area-1 for case study 1.

The transient response specifications of the LFC system for case study −1 are summarized in Table 4. Fig. 8,9 present the frequency deviation response of double area power system at Area-1 and Area-2.

**Table 4:** Transient response specifications of case study 1.

Variables	Area-1		Area-2	
	AMPC cascaded with PID	EDO-PID	AMPC cascaded with PID	EDO-PID
Overshoot	≈ 0	≈ 0	≈ 0	≈ 0
Undershoot	−0.08	−0.12	−0.083	−0.11
Settling time	≈ 4 s	≈ 7 s	4 s	10 s

**Fig. 9:**
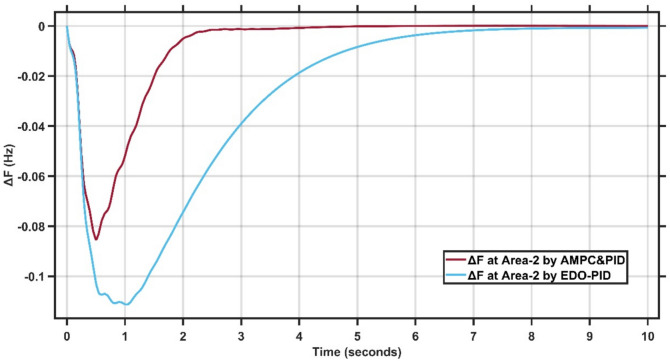
Change in frequency between AMPC cascaded with PID and EDO-PID controller at area-2 for case study 1.

From Figures 8,9, it is clear that the frequency deviation responses of the two-area interconnected power system for Area-1 and Area-2 under a step load disturbance, comparing the proposed AMPC with PID controller against the conventional EDO-PID controller. In Area-1, the proposed controller limits the maximum frequency undershoot to approximately −0.075 Hz, whereas the EDO-PID exhibits a deeper undershoot of nearly −0.12 Hz, corresponding to an improvement of about 35–40%. Similarly, in Area-2, the undershoot is reduced from approximately −0.11 Hz with EDO-PID to about −0.085 Hz using the proposed controller. Moreover, the AMPC with PID achieves a significantly faster recovery, settling within approximately 2–2.5 s in both areas, compared to around 6–7 s for the EDO-PID controller. The reduced oscillations and faster convergence in both areas confirm the superior damping capability and enhanced coordination of the proposed controller in multi-area load frequency control applications.

**Fig. 10:**
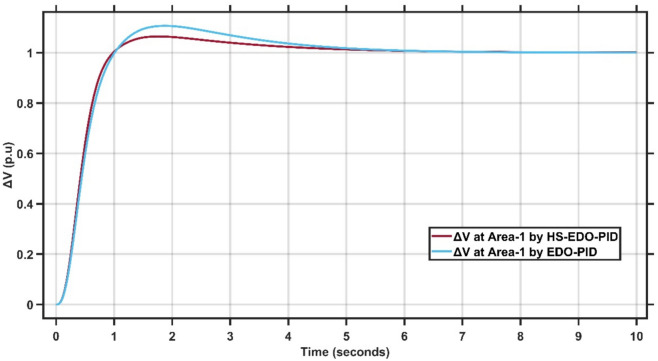
AVR response with HS-EDO-PID and EDO-PID at area-1 for case study-1.

**Fig. 11:**
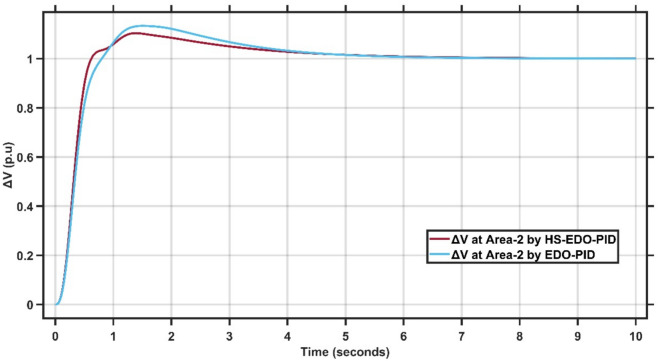
AVR response with HS-EDO-PID and EDO-PID at area-2 for case study-1.

Figures 10,11 illustrate the terminal voltage deviation response of the AVR system at Area-1 and Area-2.

From Fig. 10,11, it is clear that the terminal voltage deviation responses of the two-area interconnected power system for Area-1 and Area-2, comparing the proposed HS-EDO-PID controller with the conventional EDO-PID controller. In Area-1, the proposed controller achieves a peak overshoot of approximately 4–5%, compared to nearly 7–8% with the EDO-PID, while settling to the nominal voltage within about 3 s, whereas the EDO-PID requires approximately 5–6 s. A similar performance trend is observed in Area-2, where the overshoot is reduced from about 7% to approximately 5%, and the settling time is shortened from nearly 5–6 s to around 3 s using the proposed controller. The reduced overshoot, faster settling, and smoother transient behavior in both areas confirm the superior voltage regulation capability and improved damping characteristics of the HS-EDO-PID controller in multi-area AVR applications.

#### Robustness analysis

This section evaluates the robustness and reliability of the proposed control schemes applied to the combined LFC–AVR double-area power system. Robustness test is conducted to assess controller performance under load disturbances. For the LFC loop, the proposed cascaded AMPC–PID controller is compared with EDO-PID controller, while for the AVR loop, the proposed HS-EDO-PID controller is against the EDO-PID controller.

In this test, load disturbances are introduced in the form of step changes in load demand with magnitudes of 0.03 and 0.04 p.u on both areas. These disturbances are applied to evaluate the dynamic response of the combined LFC–AVR system under varying operating conditions. The frequency deviation response obtained using the proposed controllers are compared with response of EDO-PID controller under different conditions.

**Fig. 12:**
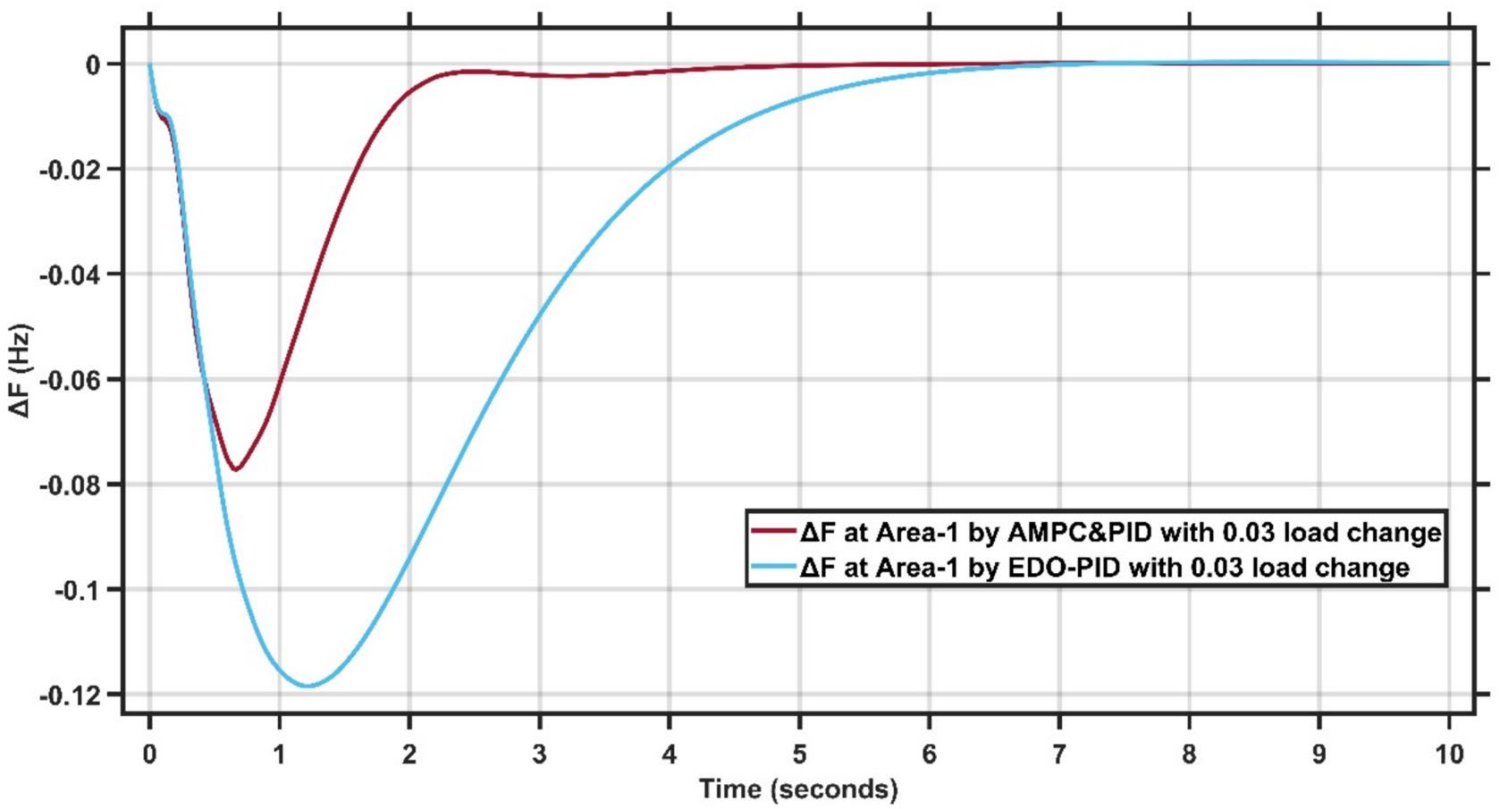
LFC response at area-1 with cascaded AMPC and PID compared to EDO-PID under 0.03 load change.

**Fig. 13:**
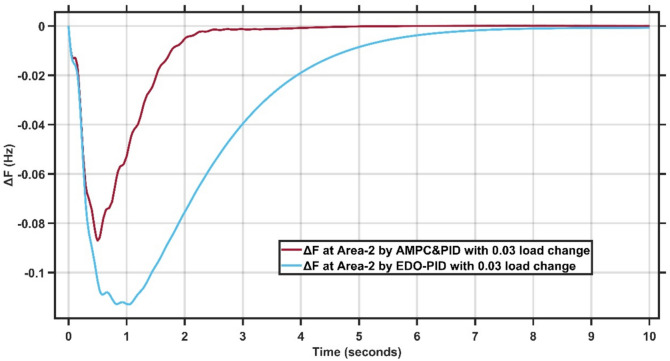
LFC response at area-2 with Cascaded AMPC and PID compared to EDO-PID under 0.03 load change.

Figures 12,13 present the frequency deviation response of double area power system at Area-1 and Area-2 with load change of 0.03 p.u.

**Fig. 14:**
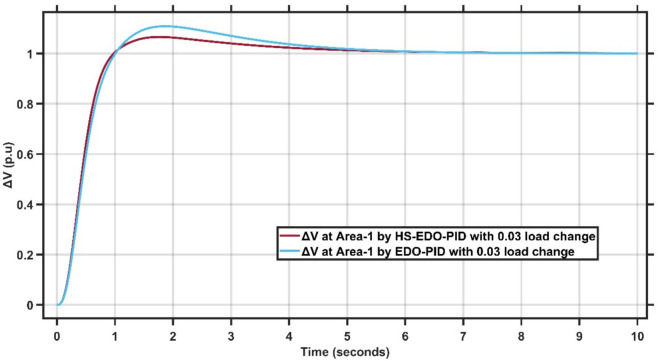
AVR response at Area-1 with HS-EDO-PID compared to EDO-PID under 0.03 load change.

**Fig. 15:**
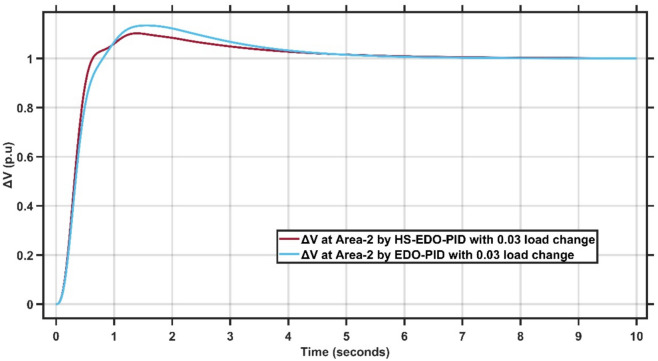
AVR response at area-2 with HS-EDO-PID compared to EDO-PID under 0.03 load change.

From Fig. 12,13, it is clear that the frequency deviation responses of the two-area interconnected power system under a 0.03 p.u. load disturbance, comparing the proposed AMPC with PID controller and the conventional EDO-PID controller. In Area-1, the proposed controller limits the maximum frequency undershoot to approximately −0.075 Hz, whereas the EDO-PID exhibits a deeper undershoot of nearly −0.12 Hz, representing an improvement of about 35–40%. Similarly, in Area-2, the undershoot is reduced from approximately −0.11 Hz with EDO-PID to about −0.085 Hz using the proposed controller. Moreover, the AMPC with PID achieves a significantly faster recovery, settling within approximately 2–2.5 s in both areas, compared to around 6–7 s for the EDO-PID controller. The reduced undershoot and faster settling in both areas confirm the superior damping performance and enhanced robustness of the proposed controller under load variations in multi-area LFC systems. Figures 14,15 illustrate the terminal voltage deviation response of the AVR system at Area-1 and Area-2 with load change of 0.03 p.u.

From Fig. 14,15, it is clear that the terminal voltage deviation responses of the two-area interconnected power system under a 0.03 p.u. load disturbance, comparing the proposed HS-EDO-PID controller with the conventional EDO-PID controller. In Area-1, the proposed controller limits the peak voltage overshoot to approximately 4–5%, compared to nearly 7–8% with the EDO-PID, while achieving voltage settling within about 3 s, whereas the EDO-PID requires approximately 5–6 s. A similar improvement is observed in Area-2, where the overshoot is reduced from about 7% to approximately 5%, and the settling time is shortened from nearly 5–6 s to around 3 s using the proposed controller. The reduced overshoot and faster voltage recovery in both areas confirm the superior damping capability and enhanced robustness of the HS-EDO-PID controller under load variations in multi-area AVR systems.

**Fig. 16:**
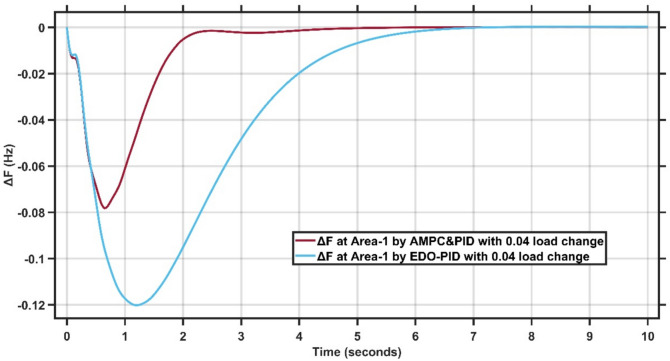
LFC response at area-1 with Cascaded AMPC and PID compared to EDO-PID under 0.04 load change.

**Fig. 17:**
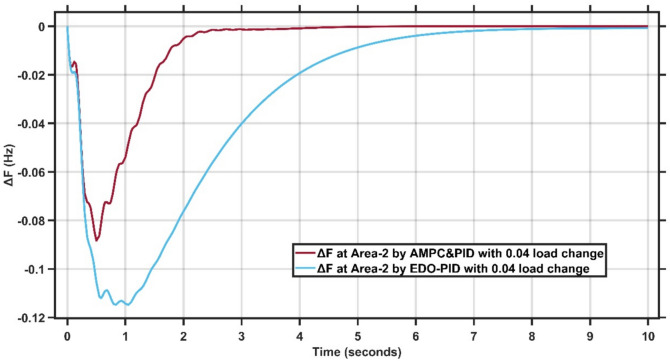
LFC response at area-2 with Cascaded AMPC and PID compared to EDO-PID under 0.04 load change.

Figures 16,17 present the frequency deviation response of double area power system at Area-1 and Area-2 with load change of 0.04 p.u.

**Fig. 18:**
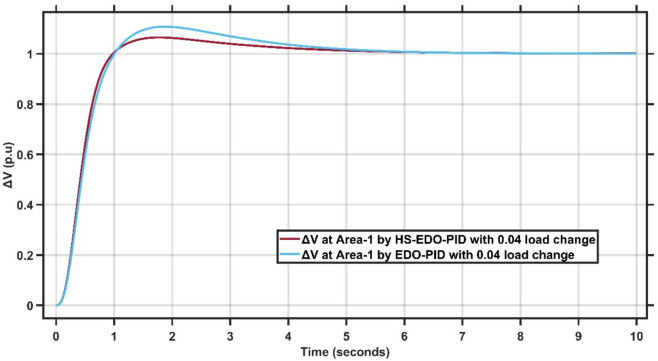
AVR response at area-1 with HS-EDO-PID compared to EDO-PID under 0.04 load change.

**Fig. 19:**
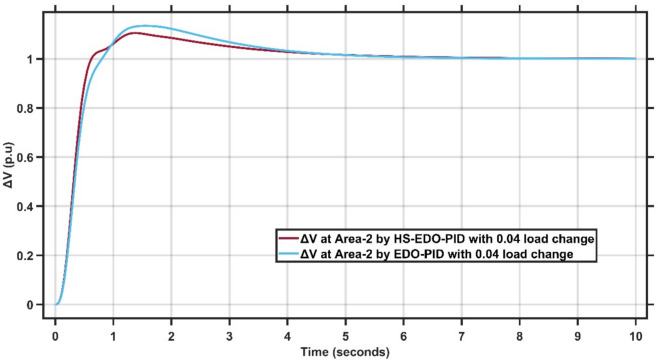
AVR response at area-2 with HS-EDO-PID compared to EDO-PID under 0.04 load change.

From Fig. 16,17, it is clear that the frequency deviation responses of the two-area interconnected power system under a relatively severe 0.04 p.u. load disturbance, comparing the proposed AMPC with PID controller with the conventional EDO-PID controller. In Area-1, the proposed controller limits the maximum frequency undershoot to approximately −0.078 Hz, whereas the EDO-PID experiences a much deeper undershoot of nearly −0.12 Hz, achieving an improvement of about 35%. Similarly, in Area-2, the undershoot is reduced from approximately −0.115 Hz with the EDO-PID to about −0.088 Hz using the proposed controller. Moreover, the AMPC with PID ensures faster frequency recovery, with settling times of approximately 2–2.5 s in both areas, compared to around 6–7 s for the EDO-PID controller. The reduced frequency deviations and faster convergence under higher load disturbances clearly demonstrate the superior robustness and damping capability of the proposed controller in multi-area LFC systems. Figures 18,19 illustrate the terminal voltage deviation response of the AVR system at Area-1 and Area-2 with load change of 0.04 p.u.

From Fig. 18,19, it is clear that Figures (18,19) illustrate the terminal voltage deviation (ΔV\Delta VΔV) responses of the two-area interconnected power system under a 0.04 p.u. load disturbance, comparing the proposed HS-EDO-PID controller with the conventional EDO-PID controller. In Area-1, the proposed controller limits the peak voltage overshoot to approximately 4–5%, whereas the EDO-PID exhibits a higher overshoot of nearly 7–8%, while achieving voltage settling within about 3 s, compared to approximately 5–6 s for the EDO-PID. A similar trend is observed in Area-2, where the overshoot is reduced from about 7% to approximately 5%, and the settling time is shortened from nearly 5–6 s to around 3 s using the proposed controller. The reduced overshoot and faster voltage recovery in both areas under higher load disturbances confirm the superior damping capability and robustness of the HS-EDO-PID controller in multi-area AVR systems.

## Conclusion

The simulation results confirm that the proposed cascaded AMPC–PID controller provides significant improvements in coordinated LFC–AVR performance compared with optimized EDO-PID controllers in both single-area and two-area power systems. In the single-area system, the proposed approach reduces the frequency overshoot from 0.8 × 10⁻^3^ to 0.3 × 10⁻^3^, decreases the undershoot from −6.2 × 10⁻^3^ to −4 × 10⁻^3^, and shortens the settling time from 8 s to approximately 6 s under a 0.2 p.u. load disturbance. In the two-area system, the AMPC–PID scheme achieves faster recovery, with settling times of approximately 4 s compared to 7–10 s for the EDO-PID controller, while reducing undershoot from −0.12 to −0.08 in Area 1 and from −0.11 to −0.083 in Area 2. The AVR responses further demonstrate reduced voltage overshoot and improved steady-state regulation under the HS-optimized PID controller. In addition, robustness analysis under large load disturbances and ±50% parameter variations shows that the proposed control strategy guarantees closed-loop stability while maintaining reliable dynamic performance under severe operating variations. These results validate that the cascaded AMPC–PID framework provides a robust, adaptive, and practically implementable solution for coordinated frequency and voltage regulation in modern interconnected power systems. Nevertheless, similar to other AMPC-based controllers, the performance depends on appropriate tuning of prediction horizons and weighting factors, and it requires sufficient real-time computational capability to support online optimization.

For future work, further validation of the proposed cascaded AMPC–PID scheme will be pursued through extended OPAL-RT hardware-in-the-loop experiments and potential laboratory-scale implementation to strengthen real-time feasibility assessment. In addition, future research will investigate nonlinear power system models and explore computationally efficient optimization techniques to further reduce the online computational burden.

## Data Availability

The datasets used and/or analyzed during the current study are available from the corresponding author on reasonable request.

## References

[1] Kundur, P. *Power System Stability and Control, * (New York, NY, McGraw-Hill, 1994).

[2] Bevrani, H., Watanabe, M. & Mitani, Y. *Power System Monitoring and Control, * (Hoboken, NJ, Wiley, 2014).

[3] Kamwa, I., Grondin, R. & Hebert, Y. Wide-area measurement based stabilizing control of large power systems. *IEEE Trans. Power Syst.* **16**(1), 136–153 (2001).

[4] Eremia, M. & Shahidehpour, M. *Handbook of Electrical Power System Dynamics, * (Hoboken, NJ, Wiley, 2013).

[5] Safari, A. & Shayeghi, H. Multi-objective optimization for combined LFC and AVR systems using evolutionary algorithms. *Energy* **186**, 115–128 (2019).

[6] Abdel-Magid, Y. L. & Abido, M. A. AGC tuning using particle swarm optimization. *IEEE Trans. Power Syst.* **18**(1), 25–31 (2003).

[7] Shayeghi, H., Jalili, A. & Shayanfar, H. A. A robust mixed H₂/H∞ based LFC of multi-area power systems. *Energy Convers. Manage.* **49**(4), 817–825 (2008).

[8] Fathy, A. & Kassem, A. M. Optimal PID controller tuning for LFC using recent optimization techniques. *ISA Trans.* **83**, 1–12 (2018).30144979

[9] Panda, S., Padhy, N. P. & Patel, R. N. Comparative performance analysis of PID controller for LFC. *Int. J. Electr. Power Energy Syst.* **33**(6), 1140–1149 (2011).

[10] Maciejowski, J. M. *Predictive Control with Constraints* (Pearson, 2002).

[11] Scattolini, R. & Colaneri, P. Hierarchical model predictive control. *IEEE Control Syst. Mag.* **29**(3), 65–76 (2009).

[12] Mohamed, A., Zaki, A. A. & Shabib, M. Adaptive MPC-based load frequency control for interconnected power systems. *IEEE Access* **8**, 174694–174707 (2020).

[13] Zhang, K. & Shi, Y. Adaptive model predictive control for a class of constrained linear systems with parametric uncertainties. *Automatica* **117**, 108974 (2020).

[14] Schwenzer, M., Ay, A. & Bürger, M. Review on model predictive control: An engineering perspective. *Int. J. Adv. Manuf. Technol.* **119**, 1531–1561 (2021).

[15] Tanaskovic, M., Fagiano, L., Smith, R. & Morari, M. Adaptive model predictive control for constrained linear systems. In *Proc. Eur. Control Conf. (ECC)* 1–6 (Zürich, Switzerland, 2013).

[16] Sun, D., Jamshidnejad, A. & De Schutter, B. Adaptive parameterized model predictive control based on reinforcement learning: A synthesis framework. *Eng. Appl. Artif. Intell.* **136**, 108996 (2024).

[17] Yu, Z. & Long, J. Review on advanced model predictive control technologies for high-power converters and industrial drives. *Electronics* **13**(24), 4969 (2024).

[18] Saadat, H. Power system analysis. Vol. 2. McGraw-hill (1999).

[19] Gupta, M., Srivastava, S. & Gupta, J. J. A novel controller for model with combined LFC and AVR loops of single area power system. *J.o.T.I.o.E.S.B.* **97**, 21–29 (2016).

[20] Amin, M. S., Mahmoud, A. A., Mekhamer, S. F. & Khamees, A. K. Enhancement of PID controller performance for a combined LFC and AVR single-and two-area model using exponential distribution optimization technique. *Sci. Rep.*10.1038/s41598-025-29137-5 (2025). 41436809 10.1038/s41598-025-29137-5 PMC12738553

[21] Nahas, N. et al. A self-adjusting adaptive AVR-LFC scheme for synchronous generators. *IEEE Transactions on Power Systems* **34**(6), 5073–5075 (2019).

[22] Kumar, V., Sharma, V., Arya, Y., Naresh, R. & Singh, A. Stochastic wind energy integrated multi source power system control via a novel model predictive controller based on Harris hawks optimization. *Energy Sour. A.* **44**(4), 10694–10719 (2022).

[23] Kumar, V., Sharma, V., Naresh, R. & Arya, Y. A novel predictive optimal control strategy for renewable penetrated interconnected power system. *Optim. Control Appl. Method.* **45**(5), 2190–2205 (2024).

[24] Ahmad, R., Arya, Y., Ahmer, M. F. & Nasiruddin, I. Enhanced frequency/voltage control in multi-source power system using CES and SSA-optimized cascade TID-FOPTID controller. *IETE J. Res.* **71**(2), 652–667 (2025).

